# Nontuberculous Mycobacteria: Ecology and Impact on Animal and Human Health

**DOI:** 10.3390/microorganisms10081516

**Published:** 2022-07-27

**Authors:** Ivo Pavlik, Vit Ulmann, Joseph O. Falkinham

**Affiliations:** 1 Faculty of Regional Development and International Studies, Mendel University in Brno, Tr. Generala Piky 7, 613 00 Brno, Czech Republic; 2 Public Health Institute Ostrava, Partyzanske Nam. 7, 702 00 Ostrava, Czech Republic; vit.ulmann@zuova.cz; 3 Department of Biological Sciences, Virginia Tech, Blacksburg, VA 24061, USA; jofiii@vt.edu

**Keywords:** saprophytic mycobacteria, saprozoic mycobacteria, potentially pathogenic mycobacteria, environmental saprophytic mycobacteria, geophagia, natural and human-engineered water systems, estuarine, hydrophobic, aerosolization, surface microlayer, biofilm formation

## Abstract

Nontuberculous mycobacteria (NTM) represent an important group of environmentally saprophytic and potentially pathogenic bacteria that can cause serious mycobacterioses in humans and animals. The sources of infections often remain undetected except for soil- or water-borne, water-washed, water-based, or water-related infections caused by groups of the *Mycobacterium (M.) avium* complex; *M. fortuitum;* and other NTM species, including *M. marinum* infection, known as fish tank granuloma, and *M. ulcerans* infection, which is described as a Buruli ulcer. NTM could be considered as water-borne, air-borne, and soil-borne pathogens (sapronoses). A lot of clinically relevant NTM species could be considered due to the enormity of published data on permanent, periodic, transient, and incidental sapronoses. Interest is currently increasing in mycobacterioses diagnosed in humans and husbandry animals (esp. pigs) caused by NTM species present in peat bogs, potting soil, garden peat, bat and bird guano, and other matrices used as garden fertilizers. NTM are present in dust particles and in water aerosols, which represent certain factors during aerogenous infection in immunosuppressed host organisms during hospitalization, speleotherapy, and leisure activities. For this Special Issue, a collection of articles providing a current view of the research on NTM—including the clinical relevance, therapy, prevention of mycobacterioses, epidemiology, and ecology—are addressed.

## 1. Long-Term Research on the Ecology of Mycobacteria

The first comprehensive piece of information on the ecology of mycobacteria came from a book by Jindrich Kazda that was published in 2000 [1]. The second extended edition of the book, published in 2009, comprehensively summarizes the information available at the time on the ecology of mycobacteria and their impact on human and animal health [2]. Two of the four co-editors of this book are deceased: Jindrich Kazda (1927–2018) and Karel Hruska (1935–2022), while Joseph Oliver Falkinham, III [3,4,5,6,7,8,9,10,11,12,13] and Ivo Pavlik [14,15,16,17,18,19,20,21] can be considered as successors in this field of research.

It can be assumed that due to changes in the environment, and especially the permanent decline in the healthy resistance of the human population (due to continuation of poor or deteriorating socio-economic conditions, a lack of availability of adequate medical care, and aging in many parts of the world), mycobacteriosis caused by nontuberculous mycobacteria (NTM) will present an ongoing issue. In addition, improvements in water treatment, namely, the introduction of widespread water disinfection (chlorination), have provided a selective environment for NTM. New findings on the ecology of NTM have revealed previously neglected or unknown facts related to their long-term survival in various components of the environment, their ability to colonize new substrates, and their interaction with humans. Finding new, abundant, or yet undiscovered sources of some clinically important NTM species will help to more effectively prevent exposure to predisposed individuals. Preventing the exposure of NTM to susceptible individuals is currently one of the most important ways to prevent mycobacteriosis.

## 2. Incidence of Mycobacterioses in Humans in the Last Three Years

The persistent incidence of pulmonary and extrapulmonary mycobacteriosis in children and adults is described herein for 2020–2022. Pulmonary mycobacteriosis is severe in individuals with a predisposition to infection and in individuals with comorbidities in the United States [22], South Korea [23,24], and China [25]. In particular, patients with cystic fibrosis are predisposed to colonization and NTM infection [26,27].

Extrapulmonary mycobacterioses have been described primarily as skin infections. The following species of NTM have been demonstrated: tattoo-associated cutaneous *Mycobacterium (M.) mageritense* infection [28], *M. marinum* hand infection masquerading as *tinea manuum* [29] and the treatment and outcome of *M. marinum* infections in 40 patients in the Netherlands [30], *M. terrae* and *M. intracellulare* tenosynovitis following hurricane relief efforts in the USA [31], and *M. smegmatis* skin infection following cosmetic procedures [32]. Two *M. abscessus* outbreaks at pediatric dental clinics were documented with more than 20 confirmed infected children with a median age of 6 years in both clinics in Atlanta, Georgia [4], and Anaheim, California [33]. Disseminated *M. szulgai* infection was diagnosed in a 25-year-old female with systemic lupus erythematosus graduating in veterinary medicine in Portugal [34].

## 3. Prevention of Exposure to Nontuberculous Mycobacteria

There are reasons for diligently seeking NTM resources for patients. The review article from Faverio et al. shows that the common aim in Italy is a comprehensive approach to the treatment and prevention of pulmonary mycobacteriosis. In addition to antibiotic treatment, it seeks the following four preventative steps: (1) avoiding exposure to environments colonized with NTM and evaluating the lifestyle and habits of patients; (2) implementing a personalized pulmonary rehabilitation plan and airway clearance techniques to improve symptoms, exercise capacity, health-related quality of life, and functional capacity in daily living activities; (3) evaluating the patient’s nutritional status, intervening to improve their health-related quality of life and control gastrointestinal side-effects during antimicrobial therapy (especially in patients with a low body mass index and a history of weight loss); and (4) managing comorbidities affecting disease outcomes, including structural lung diseases, immune status evaluation, and psychological support [35]. As the presence of NTM in showerheads has been shown to be the strongest predictor of NTM infection [36], avoiding showerhead mists in bathrooms is critical to reducing NTM exposure.

## 4. Searching for Patients’ Predisposition to Infection and Sources of Nontuberculous Mycobacteria in the Environment

*M. tuberculosis* is primarily transmitted by person-to-person contact. In contrast, NTM are mainly transmitted from the environment [1,2]. Due to this fact, careful epidemiological work is necessary to achieve a comprehensive approach to the treatment and prevention of mycobacteriosis. New comprehensive approaches have recently been chosen, especially for NTM tracing in the environment. In Portugal, for example, 359 new patients with mycobacteriosis looked for various environmental determinants and variables (humidity, mean temperature, pH, population density, and precipitation). None of the environmental determinants studied was strong enough to predict NTM geographical incidence in Portugal, except for population density (*p* < 0.001). The following parameters were significant in terms of the personal characteristics of patients: female sex (*p* < 0.001), age (*p* < 0.001), and HIV/AIDS incidence (*p* < 0.001) [37].

Sophisticated molecular biology methods have been used for the clinical and environmental epidemiology of *M. avium* complex isolates [38]. In the USA, considerable heterogeneity was found when comparing *M. avium* complex isolates from human patients with lung and disseminated infection from animals and the environment. In particular, isolates from humans and animals differed. Similarly, this has also been demonstrated in isolates from human patients and their environment [39]. In the United States, in central North Carolina, a higher incidence of pulmonary mycobacteriosis was found to be influenced by housing in hydric and acidic soils [40].

## 5. New Risks of Nontuberculous Mycobacteria in the Environment

In 2020–2021, a survey of the Czech market was conducted on plant fertilization products that contained peat and/or bat guano. We found a high incidence of NTM that contaminated up to 80–100% of these products [41]. Further, the presence of NTM in potting soils from patients with NTM lung disease [42] suggests that dust masks should be worn while gardening. The occurrence of NTM in garden substrates with peat and bat guano can therefore pose a great risk in various activities (agricultural production, hobby activities, etc.).

## 6. Benefits of Studying the Ecology of Mycobacteria

In many known and newly described mycobacterial species, we often encounter only a vague statement that their source for humans or animals is “most likely” the environment. In the “better cases”, at least the component of this environment (e.g., water, dust, or soil) in which the species “most likely” occurs is mentioned. It is not at all easy to find comprehensive knowledge about the ecology of the exact mycobacterial species that often causes serious human and animal diseases. There are few scientific teams that have systematically studied the ecology of mycobacteria. These teams usually carry out research with the intensive cooperation of physicians and veterinarians, biologists, zoologists, ecologists, climatologists, pedologists, microbiologists, biostatistics, molecular biologists, and other experts. In this way, they succeed in connecting these relatively “closed parallel highly specialized worlds”.

Humans have created ideal habitats for the NTM, particularly via plumbing. Specifically, the following characteristics of plumbing have been selected to increase the presence of NTM: surfaces that promote adherence and biofilm formation; disinfection to kill off microbial competitors; heating, which increases the numbers of NTM; and stagnation, under which NTM can grow while other pathogens may not.

Therefore, this Special Issue presents a number of results, opinions, and conclusions regarding various environmental matrices in relation to mycobacteria.

*M. fortuitum* was first described as a new species in 1938. Since then, a total of 16 members of the *M. fortuitum* group has been described. Their occurrence in aquarium freshwater and marine fish can be found in the article by Mugetti et al. [43]. Their clinical significance and occurrence in the environment is, subsequently, analyzed in two other communications published in this Special Issue [44,45]. All this information can serve readers as a good basis for further research carried out within this *M. fortuitum* group.

The occurrence of *M. chimaera* in the aquatic environment, especially in water pipes and cooling tanks of cardiology units, currently represents a significant health risk. Its capture is described in the publication by Zoccola et al., which concerns the validation of a novel diagnostic approach combining the VersaTREK™ system for recovery and real-time PCR for the identification of *M. chimaera* in water samples [46].

The cultivation evidence of NTM from heavily contaminated environmental matrices was often accompanied by the contamination of culture media. This greatly hindered the capture of different NTM species, which were present in different matrices. The publication by Ulmann et al. [47] presents the results of the modified isolation of NTM from three types of matrices (1. soil and water sediments, 2. peat and plant material, and 3. feces and animal samples), which are often contaminated with accompanying microflora. From the results, it is also worth mentioning the wide species spectrum of NTM, which helps clarify and explain the ecology of many NTM species that are not so often isolated from the environment, such as *M. algericum*, *M. bohemicum*, *M. duvalii*, *M. europaeum*, *M. fallax*, *M. goodii*, *M. hassiacum*, *M. hiberniae*, *M. malmoense*, *M. mucogenicum*, *M. nebraskense*, *M. numidimassilliense*, *M. parmense*, *M. porcinum*, *M. saskatchewanense*, *M. simiae*, and *M. timonense*. Especially surprising was the high concentration of *M. avium* ssp. *hominissuis*, *M. malmoense*, and *M. xenopi* in peat and plant material. The detection of mycobacterial DNA with qPCR from live and dead mycobacterial cells greatly accelerated and facilitated the detection of the NTM load in various environmental matrices.

Based on the results published in this Special Issue, bat guano can be considered a significant source of NTM. High culture positivity and the considerably wide spectrum of NTM species in guano originating both from caves (cave guano) and from summer colonies from attics (attic guano) is necessary in European conditions (Bulgaria, Czech Republic, France, Hungary, Italy, Romania, Slovakia, and Slovenia), considered as highly interesting. Many species from risk group two (belonging to the *M. fortuitum* group, *M. chelonae* group, *M. mucogenicum* group, and *M. avium* complex) and NTM species belonging to risk group one (belonging to the *M. terrae* complex, *M. vaccae* complex, and *M. smegmatis* complex) were isolated from guano for the first time [48].

From another publication by Hubelova et al. [49], it is possible to conclude that speleotherapy organized in caves is minimal from the point of view of the health risks posed by NTM. These results support the usefulness of speleotherapy, especially for children suffering from asthma and other chronic respiratory diseases. However, the relatively high culture positivity of NTM in spider’s webs must be considered as surprising. The isolation of NTM belonging to risk group one (*M. gordonae*, *M. kumamotonense*, *M. terrae*, and the *M. terrae* complex) and NTM belonging to risk group two (*M. avium* ssp. *hominissuis*, *M. fortuitum*, *M. intracellulare*, *M. peregrinum*, and *M. triplex*) is interesting. Indeed, dust is not very often considered to be a significant source of NTM compared to water and water biofilms [1,2].

Two reviews published in this Special Issue open novel perspectives on the ecology of NTM [50,51]. The first article written by Falkinham [50] focuses on habitats occupied by NTM (soils, sediments, dust, estuaries, and surface, ground, well, and spring water), the transmission of NTM from environmental habitats (by aerosolization, dust generation, swallowing, and surface contact), NTM characteristics contributing to environmental survival (hydrophobicity, humic and fulvic acid growth stimulation, salt tolerance, and desiccation tolerance), habitat adaptation by NTM (colony type variation and temperature tolerance in M. avium), geographic distribution of NTM in the United States, and the ecology of NTM in household and hospital plumbing.

The second review, written by Pavlik et al. [51], comes up with a novel concept of looking at NTM as sapronoses (soil-borne mycobacterioses). Although NTM are divided into two risk groups from the point of view of inducing disease in the host, practically all NTM are under certain circumstances (size and repetition of the infectious dose, immune status of the host organism, predisposing tissue injuries, iatrogenic infections after plastic and other surgical procedures, nosocomial infections, etc.) capable of causing disease. This review, therefore, introduces the reader to the history of the term “sapronosis”, first published information about NTM and sapronoses, NTM present in water, biofilms, etc., NTM present in soil, sources of NTM in plants and plant tissues (internalization of NTM in plant tissues, surface contamination of plants, and NTM of unknown natural source), geophagia in humans and animals and NTM, conditions for survival and the multiplication of NTM in the environment, environmental temperature (psychrophilic, mesophilic, thermophilic, and extremely thermophilic mycobacterial species), environmental pH, chemical conditions for survival and the multiplication of NTM in the environment (content of organic carbon in the environment, phosphorus as important element, and the impact of metallic chemical elements for NTM growth), clinical relevance of mycobacteria, and soil-borne NTM (present in soil permanently, occurring in soil temporarily during their development, surviving but not reproducing in soil, and occurring in the soil by accident).

The purpose of these articles is not to tire or bore the reader with data. These researchers show new methods of detecting mycobacteria, new perspectives on the importance of the environment in their survival and spread, and the current understanding of the ecology of mycobacteria. We, therefore, hope that these published data will also serve as a guide for further thinking about the ecology of mycobacteria.

## Data Availability

Requests on the availability of data in the published literature and correspondence should be addressed to the corresponding author.
